# Treatment of ampullary neuroendocrine tumor by Capecitabine (Xeloda®) and Temozolomide (Temodal®) 

**Published:** 2021

**Authors:** Sima Noorali, Shirin Haghighi

**Affiliations:** *Department of Hematology & Oncology, Taleghani Hospital, Shahid Beheshti University of Medical Sciences, Tehran, Iran*

**Keywords:** Neuroendocrine tumors, Vater's ampulla, Capecitabine, Temozolomide

## Abstract

Neuroendocrine tumors of the gastrointestinal tract and pancreas include a range of rare and diverse neoplasms with unique tumor biology, natural history, and clinical management. Neuroendocrine tumors of the ampulla of Vater are extremely uncommon cancers that account for only about 0.3%-1% of all gastrointestinal neuroendocrine tumors. Approximately 139 cases have been reported to date. In this paper, we describe two patients with low to intermediate grades of ampullary neuroendocrine tumors that underwent multiple courses of chemotherapy with Capecitabine (Xeloda®) and Temozolomide (Temodal®). Our two patients had a dramatic response to this regimen. According to our study, it seems that ampullary neuroendocrine tumors have behavior like pancreatic neuroendocrine tumors which requires further studies in more patients.

## Introduction

 Neuroendocrine tumors (NETs) have been detected in the esophagus, stomach, duodenum, small bowel, pancreas, colon, rectum, bronchus, ovary, testis, larynx, and biliary tract, and may differ in their response to treatment depending on their site of origin (1,2). NETs of the gastrointestinal tract and pancreas include a range of rare and diverse neoplasms with unique tumor biology, natural history, and clinical management (3). The most common of the NETs are pancreatic islet cell tumors and carcinoid tumors that represent only 2% of all gastrointestinal malignant neoplasms (4). NET of the ampulla of Vater is extremely rare and accounts for only about 0.3%-1% of all gastrointestinal NETs (5). 

## Case Reports

**Case 1**

the first case was forty years old female. The patient complained of itching and mild epigastric pain that started 2 weeks before referring to our hospital. Past medical history was unremarkable. She had a history of gastric adenocarcinoma in her father who died two years after diagnosis (the time of third debulking surgery). Physical examination revealed a generalized Jaundice. Laboratory findings showed the total and direct levels of bilirubin 17.3 and 11.3 mg/dl, respectively. In the endosonography, there was a 25*30 millimeters (mm) hypoechoic mass lesion at the ampulla and a 10-mm regional lymphadenopathy. Endoscopy showed ulcerative vegetative 22*17 mm lesion around the ampulla where biopsies were taken. Pathological examination suggested a well-differentiated NET (Grade 2) confirmed with Immunohistochemical (IHC) study which was positive for Synaptophysin, Chromogranin A (127). In this examination, Ki-67 was 4%. Computed tomography (CT) scan showed a periampullary mass (30*50 mm) resulted in extrahepatic and intrahepatic biliary dilatation. The maximum diameter of the common bile duct (CBD) was measured at 14 mm. The mass only partially touches the portal vein without a definite sign of invasion. During three months, she received three courses of chemotherapy with Capecitabine (Xeloda®- Roche company - 750 mg/m2- Two weeks of every three weeks) and Temozolomide (Temodal®- 200 mg/m2- The tenth day until the fourteenth) with a partial response based on RECIST criteria on CT scan. After three months, Whipple surgery was performed with complete R0 resection. In four years of follow-up, she was in clinical remission. But finally, liver metastasis was seen in abdominopelvic sonography. 

**Case 2**

A fifty-eight-year-old male with epigastric pain and weight loss. The result of endoscopy was gastritis with a small polyp in the antrum. Biopsy showed chronic gastritis and no true polyp structure was noted. Abdominopelvic sonography demonstrated a hypoechoic mass at the ampulla of Vater with common bile duct (CBD) and intrahepatic dilatation. CT scan revealed intrahepatic and extrahepatic bile ducts ectasia and gallbladder dilatation. Homogenous isodensity mass of the ampulla of Vater causing atrophy of the pancreas was noted. In endosonography, a mixed echoic lesion was detected at the ampulla of Vater with adhesion to the portal vein, superior mesenteric artery, and superior mesenteric vein. Biopsy of the ampulla of Vater through endoscopic retrograde cholangiopancreatography (ERCP) was performed; Pathological examination demonstrated well-differentiated NET (grade 2) of the ampulla of Vater. The IHC study was positive for Chromogranin A, Synaptophysin, CEA, and Ki-67 was 15%. In Octreoscan, a large zone of an abnormal collection of radiotracer was seen in the right upper quadrant of the abdomen, below the caudate lobe, probably the ampulla of Vater. During eleven months, he received eleven courses of systemic chemotherapy with Capecitabine (Xeloda®- Roche company- 750 mg/m2- The first day to the fourteenth day) and Temozolomide (Temodal®- 200 mg/m2- The tenth day until the fourteenth). After five courses of chemotherapy, he had a partial response to treatment and he did not accept surgery on cycle five. But eventually, on cycle eleven, the surgery was performed upon the patient's agreement. Pathological staging was carried out to complete R0 resection. In seven years of follow-up, he is still in clinical remission.

## Discussion

NETs represent 0.5% of all cancers; however, their incidence has risen in recent years, probably due to the increased frequency of healthcare examinations and improvements in diagnostic techniques (4). NETs can be part of familial cancer syndromes such as Neuroﬁbromatosis type 1 (NF1), Multiple Endocrine Neoplasia type 1 (MEN1), Von Hippel-Lindau (VHL) syndrome or Tuberous Sclerosis (TS) but most of them are sporadic (3). They have been detected in the esophagus, stomach, duodenum, small bowel, pancreas, colon, rectum, bronchus, ovary, testis, larynx, and biliary tract, and may differ in their response to treatment depending on their site of origin (1,2). Also, NETs of the gastrointestinal tract and pancreas include a range of rare and diverse neoplasms with unique tumor biology, natural history, and clinical management issues (3). The most common NETs are pancreatic islet cell tumors and carcinoid tumors that represent only 2% of all gastrointestinal malignant neoplasms (4). However, NETs of the ampulla of Vater are an extremely uncommon subset of pancreatic cancers that account for only about 0.3%-1% of all gastrointestinal NETs (5,6). Approximately 139 cases have been reported to date, and only about 20% of these reported patients are of African or Asian Pacific origin (6). 

Clinical presentations consist of jaundice, nonspecific upper abdominal discomfort, acute pancreatitis, weight loss, and upper gastrointestinal bleeding; but the leading symptom in approximately two-thirds of the patients is jaundice (7,8).

In addition to clinical findings, abdominal tomography, ERCP, endoscopic biopsy, and endosonography are also valuable in the diagnosis and detection of invasion depth; also, CT scans and octreotide scans are helpful in metastatic workup (6,7). Staining of ampullary neuroendocrine tumors (ANETs) with Chromogranin A and Synaptophysin is positive in 92-100% cases (6). ANET frequently originates from the deep mucosa or submucosa, so it cannot be easily detected in biopsy specimens and there is a high rate of false-negative or inconclusive biopsies making its diagnosis challenging till now (5,9).

ANETs are so rare, so significant questions regarding their natural history remain unanswered (10). Common sites of metastases are usually the lymph nodes and liver (9). Tumor grade and distant metastasis are the most important factors in determining survival and prognosis in ANETs. Other prognostic factors, like nodal involvement, tumor size, and resection margins, appear to be of lesser significance in predicting long-term survival (6,11). The overall five-year survival of patients with resected ANETs is 90% for well-differentiated tumors but is lesser in tumors with lower differentiation. Low-grade NETs show 5- and 10-year survival rates of 80% and 71%, respectively; in contrast, the high-grade NETs have 10-year survival rates of 15% (9). 

Optimal treatment for localized PNETs is surgical resection and for tumors that are too advanced to surgically resect, some chemotherapy regimens are effective. Recent studies examined the role of Capecitabine (Xeloda®) and Temozolomide (Temodal®) combination in well-differentiated and unresectable pancreatic neuroendocrine tumors (PNETs) and showed a high response rate (13-18). Sunitinib (Sutent®) is also a multi-targeted tyrosine kinase inhibitor that has become integrated into the treatment of progressive well-differentiated PNETs (14-16). In ampullary tumors, in most studies, the treatment of choice for neuroendocrine tumors of the ampulla of Vater is complete resection (5-7,9,12).

According to one study, a patient with a metastatic neuroendocrine tumor of the ampulla of Vater underwent chemotherapy with Sunitinib (Sutent®) and complete remission was achieved (19); and as mentioned before, Sunitinib (Sutent®) is used to for the treatment of PNETs (14-16). Our two patients had low to intermediate grades of ANETs and they received multiple courses of systemic chemotherapy with Capecitabine (Xeloda®) and Temozolomide (Temodal®) with good response to this regimen. 

Based on the above argument, and good responses of patients to Capecitabine and Temozolomide, it seems that ANETs have behavior like PNETs requiring further studies in a larger number of patients.

## Conflict of interests

The authors declare that they have no conflict of interest.
